# Structure characteristics of *Aspergillus egyptiacus* mitochondrial genome, an important fungus during the fermentation of dark tea

**DOI:** 10.1080/23802359.2018.1521308

**Published:** 2018-10-08

**Authors:** Zhenggang Xu, Liang Wu, Shiquan Liu, Yi Chen, Yunlin Zhao, Guiyan Yang

**Affiliations:** a Hunan Provincial Key Lab of Dark Tea and Jin-hua, School of Materials and Chemical Engineering, Hunan City university, Changsha, Hunan, P.R. China;; b Hunan Research Center of Engineering Technology for Utilization of Environmental and Resources Plant, Central South University of Forestry and Technology, Changsha, Hunan, P.R. China

**Keywords:** *Aspergillus egyptiacus*, mitochondrial genome, dark tea, phylogeny

## Abstract

*Aspergillus egyptiacus* is an important fungus during the fermentation process of dark tea. However, there is little study on genetic information about *A. egyptiacus.* In this study, *A. egyptiacus* was is isolated from dark tea and first sequenced. The annotation and analysis also have been done for the mitochondrial genome. The length of *A. egyptiacus* mitochondrial genome is 66,564 bp and include 41 genes. Similar to most fungus, 15 protein-coding genes (PCGs), 24 transfer RNA (tRNA) genes, 2 ribosomal RNA (rRNA) genes, and one non-coding control region (D-loop) were identified in the strand of mitochondrial genome. The GC content is 26.55% and the reconstructed phylogenetic supported the placement of *A. egyptiacus* in Eurotiomycetes clade. The mitochondrial genome information also may supply references for utilization of *A. egyptiacus.*

*Aspergillus egyptiacus* was first described by Moubasher and Moustafa in 1972 and was included in the *A. nidulans* group by most scientists (Moubasher and Moustafa 1972, Christensen and Raper 1978). *A. egyptiacus* could be used in many fields basing on its function, such as progesterone transformation (Ismail and Zohri 1994). As the unique microbial fermented tea, dark tea is fermented with microorganism during the manufacturing process. The research focus on the microorganism community is increasing in recent year for the health benefit value of dark tea. *A. egyptiacus* have been found during the fermentation process and showed playing an important role for quality determination(Guan et al. 2010, Li et al. 2017). Not according with the value of the important resource microorganism, there is little study on genetic information about *A. egyptiacus.*

*A. egyptiacus* QA was isolated from dark tea in the Hunan Provincial Key Lab of Dark Tea and Jin-hua, Yiyang, China. The fungus was cultured in liquid medium (malt extract 20 g, yeast extract powder 20 g, sucrose 30 g, and water 1000 mL) with shaking at 180 rpm at 28 °C for 7 days. Mycelia were collected by filtration on Waterman paper and placed in a mortar, liquid nitrogen was added, and the samples were crushed using a pestle. Genomic DNA from was extracted using the CTAB method and sequences were obtained and assembled together using the Illumina HISeq2500 platform (Illumina, SanDiego, CA) with paired-end reads of 100 bp (Rogers and Bendich 1994). Adapters and low-quality reads were removed using the NGS QC Toolkit (Patel and Jain 2012). The circular was generated and genome was annotated as Wu Liang’s address (Liang et al. 2018). The sequence information of *A. egyptiacus* mitochondrial genome was submitted with the accession number MH041273. Then, maximum likelihood method was employed to constructed phylogenetic tree which includes 6 Eurotiales, 4 Onygenales, 3 Hypocreales and 1 Lecanorales species respectively using MEGA7.0 software with 1000 bootstrap replication (Kumar et al. 2016).

The length of *A. egyptiacus* mitochondrial genome is 66,564 bp and include 41 genes. Similar to most fungus, 15 protein-coding genes (PCGs), 24 transfer RNA (tRNA) genes, 2 ribosomal RNA (rRNA) genes, and one non-coding control region (D-loop) were identified in the strand of mitochondrial genome. The GC content is 26.55%, with composition of A, 38.24%, T, 35.21%, C, 11.52%, and G, 15.04% respectively. The reconstructed phylogenetic tree was clustered into three clades and the results supported the placement of *A. egyptiacus* in Eurotiomycetes clade (Figure 1). The research confirms that *A. egyptiacus* has a close relationship to *Aspergillus* in molecular review. The mitochondrial genome information also may supply references for utilization of *A. egyptiacus* during dark tea fermentation process or other field.

**Figure 1.**
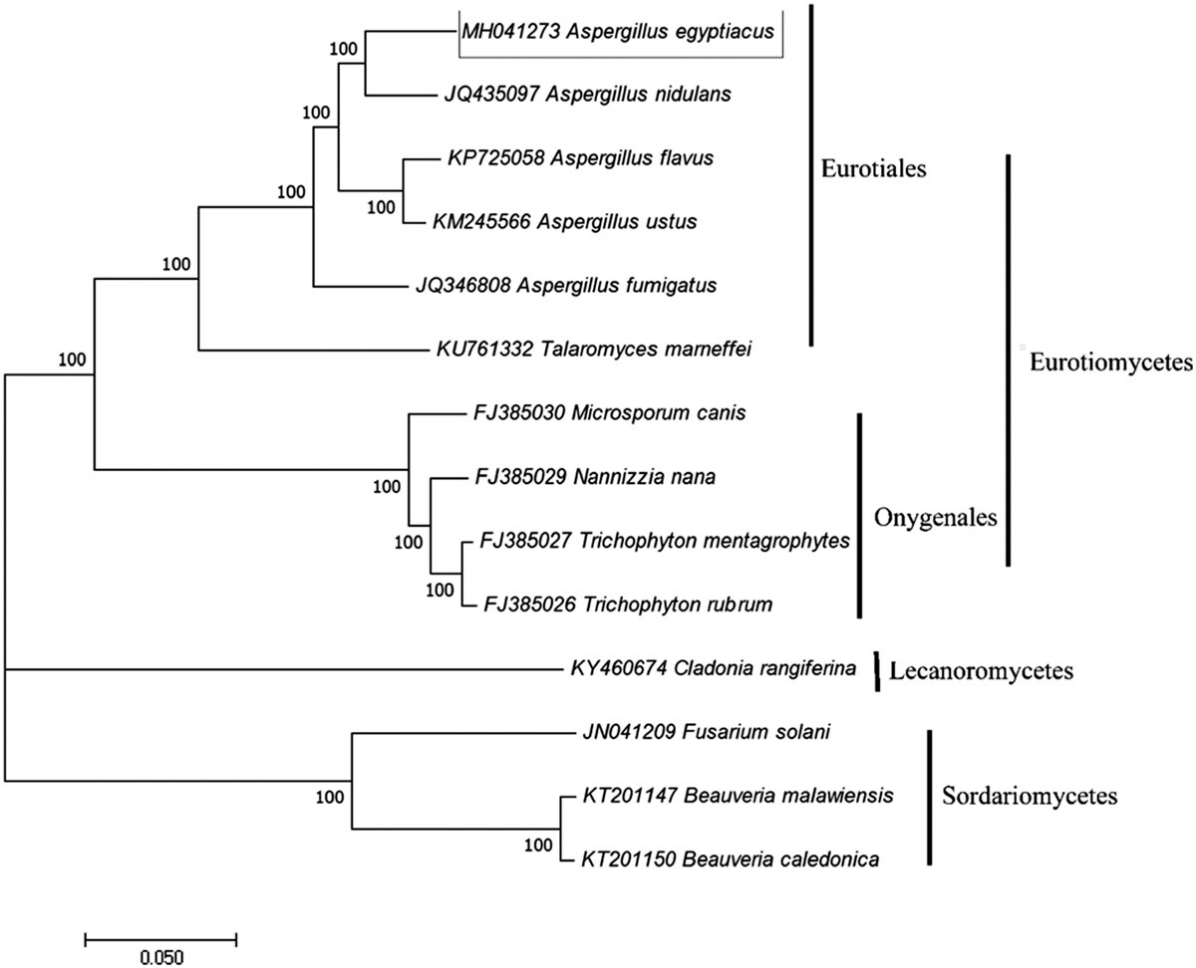
Phylogenetic tree of the relationships among *A. egyptiacus* and its related orders based on all PCGs. Branch lengths and topologies came from the maximum likelihood method (ML) analyses with 1000 bootstrap replication.
